# It only takes one to do many jobs: Amphotericin B as antifungal and immunomodulatory drug

**DOI:** 10.3389/fmicb.2012.00286

**Published:** 2012-08-08

**Authors:** Ana C. Mesa-Arango, Liliana Scorzoni, Oscar Zaragoza

**Affiliations:** ^1^Mycology Reference Laboratory, National Centre for Microbiology, Instituto de Salud Carlos IIIMajadahonda, Madrid, Spain; ^2^Group of Investigative Dermatology, University of AntioquiaMedellín, Colombia; ^3^Faculdade de Ciências Farmacêuticas de Araraquara-SP, Laboratório de Micologia Clínica, Universidade Estadual Paulista – UNESPAraraquara, Sao Paulo, Brazil

**Keywords:** amphotericin B, pore, oxidative damage, immunomodulation, fungal infection

## Abstract

“*Amphotericin B acts through pore formation at the cell membrane after binding to ergosterol*” is an accepted dogma about the action mechanism of this antifungal, and this sentence is widely found in the literature. But after 60 years of investigation, the action mechanism of Amphotericin B is not fully elucidated. Amphotericin B is a polyene substance that is one of the most effective drugs for the treatment of fungal and parasite infections. As stated above, the first mechanism of action described was pore formation after binding to the ergosterol present in the membrane. But it has also been demonstrated that AmB induces oxidative damage in the cells. Moreover, amphotericin B modulates the immune system, and this activity has been related to the protective effect of the molecule, but also to its toxicity in the host. This review tries to provide a general overview of the main aspects of this molecule, and highlight the multiple effects that this molecule has on both the fungal and host cells.

## Introduction

The control of invasive fungal infections is based on the use of antifungal drugs, being polyenes, azoles, and echinocandins the main families used in clinical practice. Among these, polyenes are the drugs that have been in use for a longer time, since they were first described in the middle of the twentieth century (Oura et al., 1955). The main polyene used as antifungal drug is Amphotericin B (AmB), which is an amphipatic macrolide. This molecule was discovered in 1950s after a broad screening of *Streptomycete* cultures that contained antifungal activity. The AmB-producing organism was isolated from a soil sample taken from the Orinoco River region (Venezuela) and was identified as *Streptomyces nodosus* (Trejo and Bennett, 1963). An intravenous presentation was introduced in the market in 1958 as a sodium deoxycholate solution (D-AmB) (Fungizone-Squibb), which forms a micellar suspension when reconstituted in glucose solution.

AmB has been used for the treatment of fungal infections and, despite the toxicity and the development of other antifungals, such as azoles and echinocandins, this drug remains as the first line treatment for severe and life threatening systemic infections such as cryptococcal meningitis and invasive zygomycosis (Saag et al., 2000; Waness et al., 2009). AmB is also effective for other mycoses such as aspergillosis, candidiasis, histoplasmosis, blastomycosis, coccidioidomycosis, sporotrichosis, fusariosis, and phaeohyphomycosis in the cases of lack of response to azoles or echinocandins. (Ellis, 2002; Davis and Porter, 2005; Metcalf and Dockrell, 2007; Chandrasekar, 2008; Gomez-Lopez et al., 2008; Muhammed et al., 2011). Additionally, AmB has activity against parasites as *Trypanosoma cruzi*, *Schistosoma mansoni*, *Echinococcus multilocularis*, and *Leishmania* spp, being the second drug of choice for the treatment for visceral leishmaniasis when antimonials fail or cannot be used (Yardley and Croft, 1999; Reuter et al., 2003; Mone et al., 2010; Paila et al., 2010). Also an amphotericin-derived drug, MS8209, has effect against HIV-1 infection avoiding virus entry to the cell (Pleskoff et al., 1995).

The D-AmB formulation has been considered the gold standard for many years and it has broad-spectrum activity. Unfortunately, this formulation is highly nephrotoxic and shows side effects as fevers, malaise, weight loss, headache, hypotension, abdominal pain, nausea, vomiting, diarrhea, normochromic normocytic anemia, and myalgia (Sabra and Branch, 1990; Meunier et al., 1991; Ringden et al., 1991; Gulati et al., 1998; Laniado-Laborin and Cabrales-Vargas, 2009). For this reason, new formulations have been introduced in the last years (Lopez-Berestein et al., 1985; Bohme and Hoelzer, 1996; Gulati et al., 1998; Rust and Jameson, 1998; Walsh et al., 1998; Dupont, 2002). The new presentations have reduced toxicity because they are lipid-carried presentations. These last formulations include a colloidal dispersion with cholesterol sulphate (CD-AmB, Amphotec), a lipidic complex with two phospholipids (LC-AmB, Abelcet) and liposomal AmB (L-AmB, Ambisome), which is integrated into true unilamellar liposomes (Veerareddy and Vobalaboina, 2004; Torrado et al., 2008). These different formulations differ in their price and in the associated toxicity. Lipid-based formulations, and in particular, L-AmB, have reduced nephrotoxicity and have superior efficacy than conventional AmB (Gulati et al., 1998; Saliba and Dupont, 2008).

## Mechanism of action of amphotericin B

**Figure 1:**
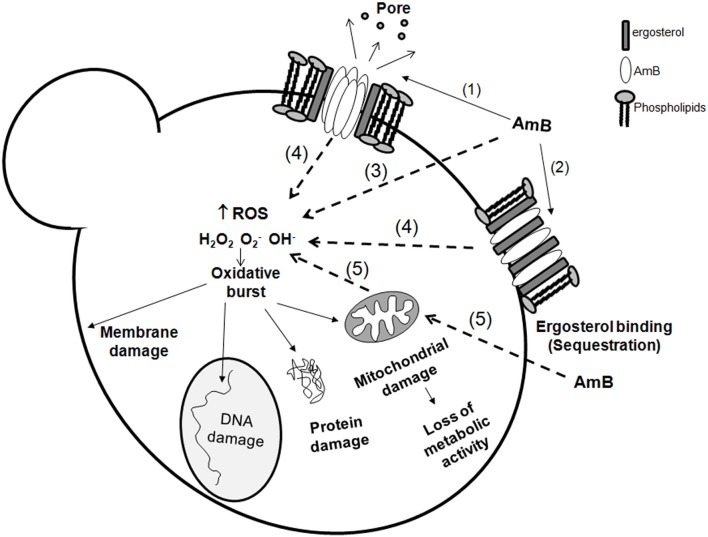
**Amphotericin B action mechanisms on fungal cells.** Amphotericin B exerts its action at different levels on the cell: membrane effects and intracellular effects. At the membrane, it can bind to ergosterol (1) and form pores, or merely induce ergosterol sequestration (2) resulting in membrane stability disruption. In the cell, AmB also induces an oxidative burst. The mechanism of this induction remains unknown, but there are several possibilities: AmB can act directly as a prooxidant (3) and induce accumulation of reactive oxygen species (ROS). However, it is also possible that this intracellular effect requires previous binding to ergosterol (4). Since ROS are natural products of the respiratory chain, it cannot be discarded that AmB influences the mitochondrial activity (5), and contribute in this way to the oxidative burst. The accumulation of free radicals has multiple deleterious effects on the essential components of the cell (membrane, proteins, DNA and mitochondria) resulting in cell death.

The mechanism of action of AmB still is not completely elucidated. AmB has effects on the fungal cell at two different levels: Binding to the ergosterol at the membrane, inducing pore formation and ergosterol sequestration, and induction of oxidative damage. In the following sections we will summarize how AmB exerts these two effects on the fungal cells, which are also summarized in Figure 1.

### Effects on the fungal membrane: pore formation and ergosterol sequestration

Early studies suggested that AmB inserts into the fungal lipid bilayer through the hydrophobic domains that bind to ergosterol. As a consequence, multimeric pores are formed, with the lipophilic polyene chains of the antifungal in contact with membrane lipids (Finkelstein and Holz, 1973; Brajtburg et al., 1990). AmB pores increase the permeability of the fungal membrane to small cations as K^+^, Ca^2+^, and Mg^2+^ promoting the rapid depletion of intracellular ions and fungal cell death (Kinsky, 1970). AmB can also bind to other sterols, such as cholesterol, but with a lower affinity (Hsuchen and Feingold, 1973).

Recently, it was proposed that AmB can exert its action through two complementary mechanisms depending on the interaction of AmB and sterols: membrane permeabilization and sterol sequestration (Palacios et al., 2011). In this sense, it has been proposed that cholesterol sequestration in the host membrane avoids macrophage–parasite interaction in *Leishmania* infection as a novel mechanism for AmB in visceral leishmaniasis (Chattopadhyay and Jafurulla, 2011).

Analytical studies have demonstrated that AmB forms two different types of pores, which differ in their substrate specificities and that are formed at different moments. Moreover, they participate differentially in the killing effect of the molecule [see seminal review in Cohen (2010) and Hartsel et al. (1994); Romero et al. (2009)]. After addition of AmB to the cells, the first type of pores that are formed are non-aqueous, which are permeable to monovalent cations and have lower permeability to monovalent anions (Ramos et al., 1996; Romero et al., 2009). Afterwards, aqueous-pores are formed, which are permeable to monovalent cations and anions and large electrolytes, such as glucose (Cohen and Gamargo, 1987; Ramos et al., 1989; Cohen et al., 1990; Cohen, 1992). The formation of pores is a very rapid process, and occurs in milliseconds. Furthermore, although AmB has affinities for both ergosterol and cholesterol (Hsuchen and Feingold, 1973), pore formation is delayed in liposomes formed with cholesterol (Mouri et al., 2008). The ergosterol and cholesterol content also determines the concentration at which AmB forms aqueous or non-aqueous pores, indicating that membrane composition has a profound effect on the AmB action (Mouri et al., 2008).

Ergosterol is required for multiple processes, such as endocytosis, vacuole fusion, and stabilization of proteins at the cell membrane (Heese-Peck et al., 2002; Zhang et al., 2010). So binding of AmB to these molecules could account for the toxic effect of the antifungal by a mechanism that involves ergosterol sequestration. This idea is supported by a recent work (Gray et al., 2012) that demonstrated that channel formation by AmB is a secondary mechanism that enhances the activity of the drug, but is not required to induce killing in the fungal cells. Using different forms of AmB that had been chemically modified, it was found that modifications that affect pore formation do not affect its antifungal activity. In this sense, it has been shown that other polyenes, such as natamycin, have antifungal effects that are not related to pore formation (Te Welscher et al., 2010).

### Induction of oxidative damage

Although it is well established that AmB binds to sterols and forms pores, there are numerous articles that indicate that increased permeability might not be the only mechanism responsible for the killing effect of the molecule. Early studies found that there was not correlation between the lethal effect of different polyenes on *C. albicans* and the degree of potassium release by the cells, suggesting that pore formation does not correlate with killing of the cells (Chen et al., 1978; Sokol-Anderson et al., 1986). This finding indicates that the formation of non-aqueous (cation-selective) pores is not enough to induce killing of the cells, and suggests that AmB elicits other killing mechanism. In this sense, it has been observed that the biological effect of AmB is very complex and depends on a variety of factors, such as the growth phase of fungi (Gale, 1974; Gale et al., 1975; Mowat et al., 2008) and the presence of oxygen (Gale et al., 1977; Sokol-Anderson et al., 1986). These data suggest that AmB action depends on metabolic factors, and indicate that the action mechanism is more complex that binding to ergosterol and pore formation. In fact, some studies argue against the idea that pore formation is the main killing mechanism. Chemical modifications of the AmB molecule that interfere with its ability to form pores do not affect its fungicidal activity (Palacios et al., 2007), which provides strong evidence that pore formation is not essential for the function of the molecule.

In agreement with the idea that AmB has other toxic mechanism than pore formation at the membrane, it has been shown that this antifungal induces oxidative stress in the cells (Sokol-Anderson et al., 1986; Haido and Barreto-Bergter, 1989; Sangalli-Leite et al., 2011). An early study demonstrated that addition of free radicals scavengers, such as catalase and/or superoxide dismutase, protects *C. albicans* protoplasts from the lytic effect of AmB (Sokol-Anderson et al., 1986). Genome-wide expression analysis confirmed that AmB, not only has an effect on the expression of genes involved in ergosterol synthesis pathway, but also induces the expression of stress genes (Liu et al., 2005), providing another evidence that AmB has pleiotropic effects in the fungal cells.

The induction of oxidative damage in the cells has been frequently reported in the literature using independent approaches. The direct production of free radicals by AmB has been measured using probes that emit fluorescence after being attacked by the free radicals, such as dihydrofluorescein diacetate or dihydrorhodamine 123 (Phillips et al., 2003; Sangalli-Leite et al., 2011). Lipid peroxidation, protein carbonylation, and apoptotic-like phenotypes (such as DNA fragmentation and anexin V staining) have been also used as indicators of oxidative stress generated by AmB in fungal cells (Phillips et al., 2003; Mousavi and Robson, 2004; Blum et al., 2008; Al-Dhaheri and Douglas, 2010; Sharma et al., 2010; Sangalli-Leite et al., 2011).

The role of oxidative damage in the antifungal effect of AmB is still unknown, but different studies suggest that this mechanism participates in this effect. A recent study demonstrates that killing of *C. neoformans* cells, measured by propidium iodide uptake, occurs after the induction of an oxidative burst. The mechanism by which AmB induces oxidative burst in the cells remains unknown. Several studies demonstrate that AmB can autooxidize, which suggests a mechanism by which AmB induces oxidative stress in the cells (Lamy-Freund et al., 1985; Sokol-Anderson et al., 1986). On the other hand, it has been demonstrated that AmB can also act as an antioxidant similar to carotenoid and retinoids (Osaka et al., 1997).

AmB induces oxidative damage in organisms others than fungi (Haido and Barreto-Bergter, 1989). Moreover, this feature of the antifungal has been related to the reduction in virulence observed in some parasite infection models. For example, AmB does not have a direct effect on development of the miracidia (larval stages) and sporocyst of the parasite *Schistosoma mansoni*, but it decreases its infectivity through a process linked to the oxidative damage induced by the antifungal that impaired the response of the parasite during infection (Mone et al., 2010).

## Mechanisms of resistance to AmB

Acquired resistance to AmB is very low despite its widespread use. Secondary resistance has been described in *C. tropicalis*, *C. parapsilosis*, *C. lusitanie*, and *C. haemulonii* (Powderly et al., 1988; Ellis, 2002). In contrast, in the last years, there has been an increase in the incidence of infections caused by fungi intrinsically resistant to AmB, such as *A. terreus*, *Fusarium* spp, and *Scedosporium prolificans* (Cuenca-Estrella et al., 1999; Sutton et al., 1999; Khan et al., 2007; Rogasi et al., 2007).

Resistance to this antifungal is achieved in different ways. Decrease in ergosterol content results in resistance to this compound (Kim and Kwon-Chung, 1974; Kim et al., 1974; Woods et al., 1974; Safe et al., 1977; Drutz and Lehrer, 1978; Merz and Sandford, 1979; Kreiner et al., 1993; Kelly et al., 1994; Currie et al., 1995; Ghannoum and Rice, 1999; Walsh et al., 2003; Vandeputte et al., 2007). Most of these studies showed alterations in the ergosterol synthesis pathway and accumulation of sterol intermediates. Moreover, in biofilms (which are microbial populations that grow attached to a surface and have reduced susceptibility to antimicrobials), resistance to AmB has been associated not only to a decrease in the ergosterol content, but also to changes in the cell wall (Khot et al., 2006). Since azoles inhibit ergosterol synthesis, cross resistance between azole and AmB has been described in the literature (Sud and Feingold, 1983; Kelly et al., 1996, 1997; Nolte et al., 1997; Sanglard et al., 2003).

However, other studies did not find a correlation between ergosterol content and susceptibility to AmB (Joseph-Horne et al., 1996a,b; Dannaoui et al., 2000). In agreement, it has been shown that pre-exposure of *C. albicans* cells to fluconazole can protect the yeasts from AmB treatment, and this effect is still present when ergosterol is added to the medium, suggesting that this resistance phenotype does not depend on ergosterol (Vazquez et al., 1998). Interestingly, it has been recently shown that subinhibitory concentrations of fluconazole induce a response in yeast that confer resistance to oxidative and nitrosative stress (Arana et al., 2010), which supports the idea that adaptation to oxidative stress can result in AmB tolerance. Resistance to AmB has been also studied using genome-wide expression analysis in *C. albicans* (Barker et al., 2004). This work demonstrated that resistance to AmB and fluconazole was associated, not only with an increase in the expression or *ERG* genes, but also with the induction of stress genes such as catalase, and reduction of mitochondrial enzymes, such as cytochrome c oxidase and acetyl CoA synthetase, suggesting that resistance to AmB could be associated to a decrease in mitochondrial activity and reactive oxygen intermediates (ROIs) production.

A strong support for the role of oxidative damage in the antifungal activity of the drug is provided by the relationship between resistance to AmB and to oxidant stress. This was first described in *C. albicans*, where it was observed that resistant strains to AmB had reduced susceptibility to H_2_O_2_ (Sokol-Anderson et al., 1988). In this work, resistance to AmB and H_2_O_2_ correlated with increased catalase activity. Another evidence of the importance of the oxidative damage was provided in the filamentous fungi *Aspergillus terreus*, which is considered intrinsically resistant to AmB. This fungus has similar ergosterol levels than a susceptible species, such as *A. fumigatus* (Dannaoui et al., 2000; Blum et al., 2008). However, AmB did not induce lipid peroxidation in *A. terreus*, suggesting that this fungus has an induction in antioxidant mechanisms. In agreement, catalase activity in *A. terreus* was significantly higher in this fungus than in *A. fumigatus* (Blum et al., 2008).

The respiratory chain in the mitochondria plays a key role in the production of free radicals in the cells because these molecules are subproducts of the respiration. So it is tempting to correlate the effect of AmB with the mitochondrial activity. Little is known about this correlation, but it has been demonstrated that disruption of respiratory function results in increased resistance to AmB in *C. albicans* (Geraghty and Kavanagh, 2003). This finding is very relevant, especially because the mitochondria is not only required for the accumulation of free radicals, but also because it is necessary for ergosterol biosynthesis, so changes in mitochondrial activity can influence the antifungal activity of AmB at multiple levels.

## AmB as a molecule with immunomodulatory properties

Antifungal drugs are derived from natural compounds with complex structure, and many of them have other effects than growth inhibition or killing of fungi. In this sense, antifungals have inmmunomodulatory properties [see review in Ben-Ami et al. (2008)]. AmB is a good example about this type of drugs. Besides the direct action on the fungal cell, several studies have shown that AmB has a potent immunomodulatory effect on the host cells. This has been demonstrated *in vitro* in different cellular lines from human and murine models, such as polymorphonuclear neutrophils (PMNs), macrophages, NK cells, T, B, and tumoral cells, but also *in vivo* in animal models. The immunomodulatory properties offer an alternative action mechanism for this antifungal by enhancing the immune response of the host. But at the same time, this effect has been related to toxicity associated to this drug. In the following sections, we will briefly review the main immunomodulatory properties of AmB.

### Immunomodulation *In vitro* and *In vivo*

**Figure 2:**
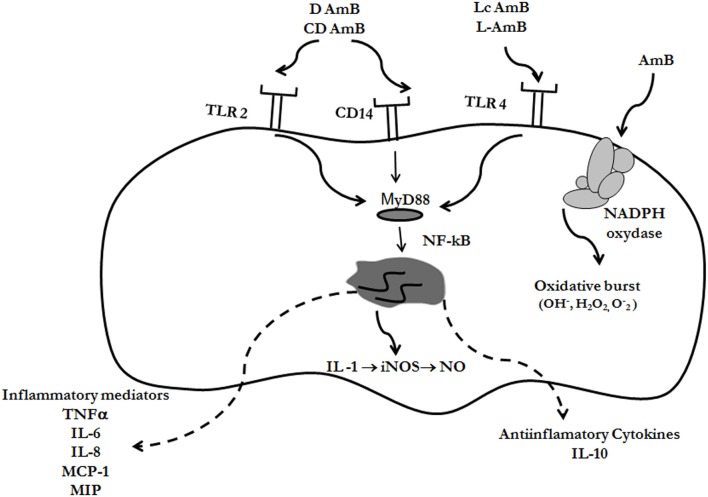
**Immunomodulatory effects of AmB.** Different formulations of AmB can bind to Toll-like receptors (TLR-2 or TLR-4) or CD14, resulting in immunomodulation of the cell. The signal is transduced through the adaptor protein MyD88, and as a final effect, NF-kB is activated and translocated to the nucleus. In this way, cytokines are expressed, which can be pro- or anti-inflammatory, depending on the AmB formulation, and receptors involved (see text for further details). AmB also induce the accumulation of free radicals (reactive oxygen intermediates, ROIs, and nitric oxide, NO) through induction of NO synthase and NADPH oxidase

Multiple studies performed *in vitro* and *in vivo* have demonstrated that AmB has an effect on the host, not only in the presence of the pathogen, but also when uninfected cell lines or animals are treated with the antifungal. AmB stimulates transcription and production of multiple mediators of the immune system (such as cytokines, chemokines, and prostaglandins) and ICAM-1 in murine and human cells (Borden and Leonhardt, 1976; Sculier and Body, 1991; Cleary et al., 1992; Louie et al., 1994; Saxena et al., 1999; Rogers et al., 2000; Sau et al., 2003; Camacho et al., 2004; Simitsopoulou and Roilides, 2005; Simitsopoulou et al., 2005). Moreover, this antifungal upregulates the expression of genes involved in angiogenesis (Lin et al., 2009). AmB also induces the accumulation of nitric oxide (NO) (Mozaffarian et al., 1997) and ROIs (Wilson et al., 1991). Most of these effects are summarize in Figure 2. In endothelial activated cells, AmB increases iNOS expression mediated by endogenous IL-1 and, in consequence, AmB augments the production of NO, which plays important role in vasodilation and protection against pathogens (Suschek et al., 2002).

The immunomodulatory properties and the proinflammatory effect induced by AmB have been associated with protective effects during infection. AmB enhances the antifungal activity of PMN and pulmonary alveolar macrophages against conidia and/or hyphal phase of *A. fumigatus* (Roilides et al., 2002). Similar results were published with murine peritoneal macrophages pretreated with IFN-γ and different doses of AmB. In this case AmB induced the production of NO, TNF-α, and IL-1, that enhanced the anticryptococcal activity of these cells (Tohyama et al., 1996).

Macrophage oxidative burst, leading to O_2_^−^ release, is activated *in vivo* after intraperitoneal injections of recombinant IFN-γ and TNF-α or AmB (Wolf and Massof, 1990). Moreover, when AmB was combined with IFN-γ, a synergic effect was observed, suggesting that IFN-γ may serve as a useful adjuvant during the treatment of intracellular fungal infections.

AmB also produces oxidative burst in macrophages following stimulation with phorbol myristate acetate. This effect was related to the binding to the antifungal to the membrane that could in turn induce conformational changes that activate membrane enzymes involved in the induction of oxidative burst, such as NADPH oxidase (Chapman and Hibbs, 1978; Wilson et al., 1991). AmB also has a cooperative effect with IFN-γ in enhancing the candidastatic activity of the macrophages through a process that involves the accumulation of ROIs (Coste et al., 2002). However, the same authors also noticed that AmB had a cooperative effect with IL-13, but this effect was independent of ROIs, indicating that AmB can activate macrophages in different ways.

The outcome of systemic and mucosal fungal infections depends on the Th response of the host. Th1 response (which depends on proinflamamatory cytokines TNF-α, IFN-γ, IL-1, IL-6) leads to resistance because it primes the immune system with macrophage inflammatory activation and superoxide and NO production. In contrast, a Th2 response (IL-10, IL-4, IL-2, IL-13, and IL-5) is associated with susceptibility to infection and disease enhancement (Puccetti et al., 1995; Romani and Howard, 1995). To evaluate the effect of AmB on the Th cell response, mice with disseminated or gastrointestinal candidiasis were treated with antifungal alone or in combination with an IL-4 antagonist, and the production of IFN-γ (Th1) and IL-4 (Th2) was evaluated. AmB induced a protective Th1 response with concomitant IL-4 depletion (Cenci et al., 1997). Similar results were observed in Balb/c mice infected with *A. fumigatus* spores and treated with AmB (Saxena et al., 1999). In agreement, it was described that AmB induces up-regulation of IL-1β and TNF-α in mouse kidney (Falk et al., 2005). The idea that AmB exerts part of its effect through immunomodulation was supported by the fact the antifungal shows a defect in the protection when mice receive neutralizing TNF-α antibodies (Louie et al., 1995).

Since changes in the immune response could have profound consequences in the host, the immunomodulatory properties of AmB can explain some of the secondary effects of the molecule. For example, the increase in proinflammatory cytokines has been correlated with the toxicity of AmB (Chia and McManus, 1990; Cleary et al., 1992; Arning et al., 1995; Shadkchan et al., 2004). In addition, direct renal toxicity has been described by the induction of apoptosis and alterations in the expression of the constitutive NO synthase (Suschek et al., 2000; Falk et al., 2005; Yano et al., 2009).

AmB has been occasionally described to have immunosupressor effects. In the human THP-1 monocytic cell line, pretreatment with AmB and challenge with *A. fumigatus* conidia results in reduced expression of TNF-α (Choi et al., 2010). Also Becker et al. observed decrease of IL-6, macrophage inflammatory protein (MIP-2), and monocyte chemoattractant protein (MCP-1) in neutropenic rats with invasive pulmonary aspergillosis treated with AmB (Becker et al., 2003).

### Mechanisms by which AmB induces immunomodulation

The mechanism by which AmB produces immunomodulation and induction of ROIs and NO is not fully elucidated. As stated above, AmB binds to the mammalian membrane because it presents affinity to cholesterol, and in this way, it could induce conformational changes that activate the NADPH oxidase enzyme (Chapman and Hibbs, 1978; Wilson et al., 1991) (Figure 2). But the mechanism that better explains the immunomodulatory effects of AmB is mediated through the Toll-like receptor (TLRs) signaling pathway (Figure 2). TLRs are members of a conserved family of mammalian receptors that recognize microbial products, being TLR2 and TLR4 the best characterized. TLR2 presents affinity for Gram-positive bacteria, peptidoglycan, lipoteichoic acid, and zymosan, whereas TLR4 ligands include LPS from Gram-negative bacteria, Taxol, and *Cryptococcus neoformans* capsular polysaccharide (Shoham et al., 2001; Janeway et al., 2005). AmB can bind to TLR, resulting in cytokine and chemokine release. Binding to TLR2 has been associated to release of proinflammatory cytokines, while binding to TLR4 produced release of anti-inflammatory (Bellocchio et al., 2005). Binding of AmB to the TLRs triggers polymerization of receptors which results in recruitment of the adaptor protein, MyD88. This signaling produces the nuclear translocation of NF-kB, which induces the expression of genes involved in macrophage activation. In addition, AmB also exerts its immunomodulatory effect through CD14 (Trajkovic et al., 2001; Sau et al., 2003), which is a receptor that activates the TLR signaling pathway after binding to LPS.

### Effect of the AmB formulations on the immunomodulatory properties

The immunomodulatory properties of AmB depend on the clinical presentation used in the treatment. In a study using plasma of patients treated with different presentations, it was found that D-AmB and L-AmB increased TNF-α, IL-6, and IL-1-RA, but this effect was not observed when patients were treated with CD-AmB (Arning et al., 1995). In human monocytes D-AmB and CD-AmB induced up-regulation of inflammatory cytokines such as IL-1, TNF-α, monocyte chemotactic protein 1 (MCP-1), and macrophage inflammatory protein 1 (MIP-1), while LC-AmB lipid complex and L-AmB down-regulated or had no effect on the gene expression of these proinflammatory cytokines (Simitsopoulou et al., 2005). Moreover, D-AmB is more effective than LC-AmB in enhancing PMN oxidative activity and D-AmB induced higher expression of CD11b/CD18 integrin (Mac-1) (Sullivan et al., 1992). On the other hand, using antibody arrays, both D-AmB and CD-AmB induced proinflammatory cytokines in the THP-1 monocytic cell line (IL-8, TNF-γ, MCP-1, and RANTES) while LC-AmB and L-AmB had no effect (Turtinen et al., 2004). This difference between the AmB formulations can be explained by the type of TLR to which the different AmB presentations bind. D-AmB binds to TLR2, which induces a pro-inflammatory response, in contrast to L-AmB which induces anti-inflammatory effect after binding to TLR4 in PMNs (Bellocchio et al., 2005).

## Conclusions and future perspectives

AmB is still an enigmatic molecule, and although it has been vastly used for the treatment of fungal infections during decades, there are still aspects about its action mechanism that remain unknown. Although the first studies demonstrated that this drug binds to sterols and in particular, ergosterol, and forms pores at the membrane, it has been also shown that AmB induces oxidative damage in the cells. There are contradictory data about the importance of these mechanisms. The fact that resistance to this antifungal correlates with different mechanisms, such as a reduction in ergosterol content or induction of antioxidant enzymes indicates that most probably both are required for the killing effect of the molecule. At the moment, it is not possible to know if these mechanisms are related or are independent, although a tempting hypothesis is that binding to ergosterol is necessary not only for pore formation, but also for the induction of oxidative damage. Further studies are required to clarify the importance of each mechanism. In addition, the induction of different killing mechanisms is in agreement with the fact that AmB is the antifungal drug with a stronger fungicidal activity. To make the situation more complex, AmB has also strong immunomodulatory properties, and in particular, it induces proinflammatory responses. This effect has been associated with protective effects, but also with the toxicity. The immunomodulatory properties of the antifungal open many questions about how AmB acts during infection, not only on the pathogen, but also on the host. This issue is of particular interest because patients affected by fungal infections are immunocompromised. So it is important to consider that AmB may have different effects on patients with different immunological states and therefore, the antifungal treatment could have unpredicted consequences in the outcome of the disease. Although AmB is one of the most effective treatments for fungal infections and secondary clinical resistance remains low, there is an increase in the incidence of pathogens that have intrinsic resistance to this antifungal, such as *Trichosporon* spp, *A. terreus* and *Scedosporium prolificans*, so more studies are required to understand the basis of intrinsic resistance and to provide an efficient strategy for the management of these infections.

### Conflict of interest statement

The authors declare that the research was conducted in the absence of any commercial or financial relationships that could be construed as a potential conflict of interest.
